# Viral metatranscriptomic approach to study the diversity of virus(es) associated with Common Bean (*Phaseolus vulgaris* L.) in the North-Western Himalayan region of India

**DOI:** 10.3389/fmicb.2022.943382

**Published:** 2022-09-21

**Authors:** Shahjahan Rashid, Farhana Wani, Gowhar Ali, Tariq A. Sofi, Zahoor Ahmed Dar, Aflaq Hamid

**Affiliations:** ^1^Department of Plant Pathology, Sher-e-Kashmir University of Agricultural Sciences & Technology of Kashmir, Srinagar, India; ^2^Department of Genetics and Plant Breeding, Sher-e-Kashmir University of Agricultural Sciences & Technology of Kashmir, Srinagar, India

**Keywords:** next generation sequencing, BCMV, BCMNV, ClYVV, multiplex PCR, recombination, phylogenetic analysis

## Abstract

Plant viruses are a major threat to legume production worldwide. In recent years, new virus strains have emerged with increasing frequencies in various legume cropping systems, which demands the development of cutting-edge virus surveillance techniques. In this study, we surveyed the common bean fields of Kashmir valley for virus infection using a total of 140 symptomatic and non-symptomatic leaf samples collected from different locations. The genetic diversity of viruses was examined by high-throughput sequencing (HTS) with three viruses being identified, namely, Bean Common Mosaic Virus (BCMV), Bean Common Mosaic Necrosis Virus (BCMNV), and Clover Yellow Vein Virus (ClYVV). BCMNV and ClYVV are new reports from India. *De novo* assembly of transcriptome constructed near-complete genomes of these viruses. RT-PCR results confirmed the presence of these viruses with an emerge incidence of 56. 4% for BCMV, 27.1% for BCMNV and 16.4 for ClYVV in the valley. Several samples were found to contain multiple virus infections with BCMV being the most predominant. Recombination events were detected in the genomes of BCMV and ClYVV, but not BCMNV. Phylogenetic and pairwise identity matrix evidence suggests viral import from multiple countries. Our results demonstrate that HTS followed by multiplex PCR assay is a simple, rapid, and reliable approach for simultaneous diagnosis of plant viruses.

## Introduction

Plants are vulnerable to various disease-causing pathogens, namely, fungi, bacteria, viruses, and nematodes. Viruses particularly can have a significant negative impact on the quality and yield of various agricultural crops (Jones et al., 2017). Every year crop losses due to viral diseases cost billions of dollars (Rubio et al., 2020). Among the different pulse crops susceptible to viruses, the common bean (*Phaseolus vulgaris L*.) is the most widely grown legume crop worldwide, namely, in India. Annually, around 31 million tonnes of dry bean grains are produced worldwide and India is among the countries with the largest production of dry beans (FAOSTAT., 2021). Common bean is locally called “Rajmash” and is an important part of the regional diet as it has high nutritional attributes. Being high in minerals, fibers, and a cheap source of protein, beans are used instead of meat in developing and under-developed countries which makes it a “grain of hope” for poor communities and is also called “Poor Man's Meat” (Zargar et al., 2017; Celmeli et al., 2018; Choudhary et al., 2018; Nadeem et al., 2021).

Common bean in the natural environment is threatened by an attack of multiple pathogens, especially viruses. It has been found susceptible to more than 70 viruses (Morales, 1986) and more than 30 viruses have been well characterized (Matthews, 1982; Hall, 1991; Loebenstein and Thottappilly, 2004). The majority of plant viruses have RNA as their genetic material and some have DNA (Kesanakurti et al., 2016). The most devastating RNA viral pathogens that infect common bean are Bean common mosaic virus (BCMV) and Bean common mosaic necrosis virus (BCMNV) in the genus potyvirus, family potyviridae (Worrall et al., 2015). Other examples of well-known common bean viral pathogens with genome as RNA are Bean yellow mosaic virus (BYMV), Clover yellow vein virus (ClYVV), Soybean mosaic virus (SMV), Cucumber mosaic virus (CMV), and Cowpea aphid-borne mosaic virus (CABMV) Potyvirus. Some DNA viruses are also reported to infect common beans such as Bean golden mosaic virus (BGMV), and Bean golden yellow mosaic virus (BGYMV). These viruses readily transmit via seed or insect vectors or by both and cause significant yield losses to the crop (Garrido-Ramirez and Sudarshana, 2000; Bonfim et al., 2007; Larsen et al., 2008; Worrall et al., 2015; Wainaina et al., 2019).

International trade, rapid climate change, and ability of viruses to rapid evolution have resulted in the frequent appearance of new viruses (Rubio et al., 2020). In recent years, new sophisticated technologies have opened new avenues in the detection of viruses and viroids. HTS is a broad-spectrum diagnostic screening approach employed for plant virus detection in 2009 (Adams et al., 2009; Kreuze et al., 2009). Since then various nucleic acid inputs have been used for library preparation using ribosomal RNA-depleted total RNA, small RNAs, total RNA, mRNA, and double-stranded RNA for HTS. The use of HTS has made it possible to detect not only known viruses but also novel/non-target viral pathogens for which other diagnostics are not available yet (Xu et al., 2019). The HTS is a broad-spectrum diagnostic tool in which prior knowledge of viral pathogens is not needed, as it enables the sequencing of all the genomic material in the sample (Adams et al., 2009; Maree et al., 2018; Gaafar et al., 2020). Unlike traditional virus diagnostic approaches which are based on observational, serological, and molecular methods which target only known pathogens and depend on prior knowledge of the pathogens being tested (Gaafar et al., 2020).

In addition, a mixed viral infection of crop plants occurs commonly in nature as a consequence of successive vector inoculations (Rubio et al., 2020). During these conditions, it becomes difficult to understand the disease etiology. HTS technique has provided us with a powerful tool to reveal the etiology of latent infections and unknown diseases and for the detection and identification of virome of infected plants (Barba et al., 2014).

Early identification of the viral pathogen is essential for limiting the spread of viral diseases as well as combating their negative effects on the yield of agricultural crops (Akinyemi et al., 2016). Accurate diagnosis and proper identification are the basic steps for managing any viral disease (Chiquito-Almanza et al., 2017). Using HTS 15 viruses were identified from common beans, namely, Southern bean mosaic virus (SBMV) and Tomato leaf curl Uganda virus for the first time in Tanzania (Mwaipopo et al., 2018). In Kenya, two cryptic double-stranded RNA viruses Phaseolus vulgaris endornavirus 1 (PvEV-1) and Phaseolus vulgaris endornavirus 2 (PvEV-2) beside Bean common mosaic necrosis virus (BCMNV), and Cucumber mosaic virus (CMV) were identified by using Illumina RNA-seq (Mutuku et al., 2018).

Although common bean is grown in several regions of India, there are only a few reports where attempts have been made to detect bean virus pathogens. Also, these studies were based on classical virus detection techniques. In this study, we used the viral metatranscriptomic approach by using Illumina high-throughput RNA sequencing to profile the virome of common bean that showed diverse virus-like symptoms. This is the first virome analysis of common beans from India. We identified three viruses by using a bioinformatics pipeline; two viruses are the first reports from India in common bean. Our study will be helpful in the production of disease-free seed stock in certification programs and the development of disease management strategies.

## Materials and methods

### Survey and collection of samples

**Figure 1:**
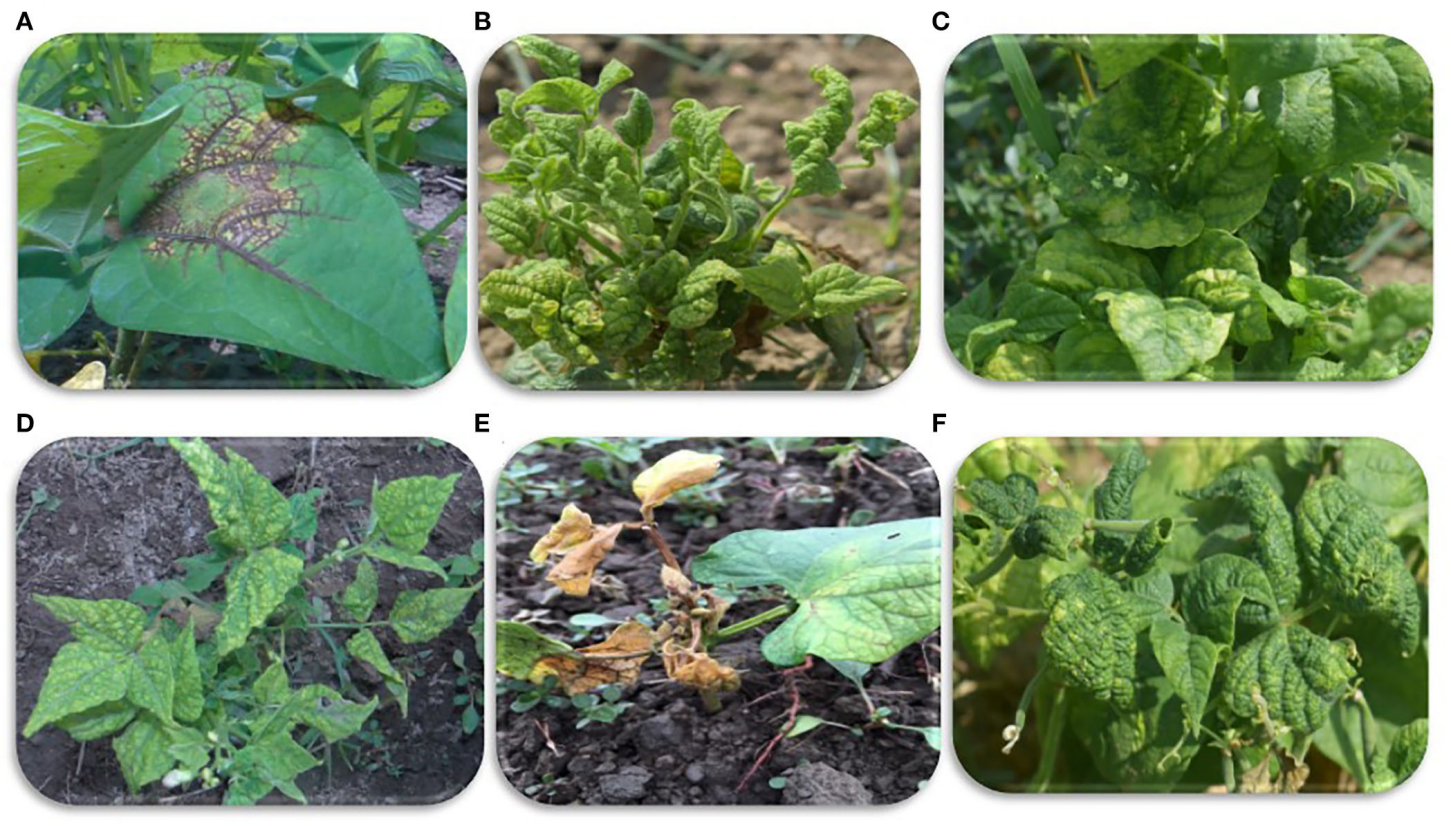
Viral symptoms on common beans observed during sample collection. **(A)** Veinal necrosis. **(B,D,F)** Mosaic and severe leaf deformities, **(C)** mosaic and puckering, and **(E)** mosaic and leaf necrosis.

In the early summer of 2020, a survey was conducted in Jammu and Kashmir, India, to determine the viral community present in common beans. The area was divided into three regions South, Central, and North Kashmir. In each region, 5–10 fields of common bean were selected randomly for the collection of leaf samples. Common bean leaf samples collected during field surveys exhibited mosaic, necrosis, venial necrosis, puckering, leaf distortion, stunted growth, and upward and downward curling symptoms (Figure 1). Samples were collected from every selected field in RNAlater (Thermo Fisher Scientific Inc.). A total of 140 leaf samples of common bean were collected from Kashmir valley (**Table 3**). Some asymptomatic samples were also collected. Collected samples were stored at−80 C prior to further analysis.

### RNA isolation, library preparation, and sequencing

Total RNA was extracted from leaf samples by grinding the samples in liquid nitrogen using the mortar pestle or TissueLyser and the fine tissue powder was immediately transferred into the Eppendorf tube 1.5μl and placed in liquid nitrogen. Total RNA was isolated from leaf samples using TRIzol reagent (Thermo Fisher Scientific Inc). The pellets were centrifuged and washed following RNA precipitation, wash was discarded and pellets were air dried and then dissolved in 30–50μl of nuclease-free water. RNA samples were checked for their quality and quantity using a NanoDrop 2000c UV-vis spectrophotometer (Thermo Fisher Scientific Inc.) and Agarose gel electrophoresis (1% Agarose Gel) containing TAE buffer. Out of 140 samples, RNA isolated from 14 samples (four from South, four from North, and six from Central Kashmir) were pooled to form a single sample and sent for virome analysis at Nucleome Biotech, Hyderabad, India for sequencing total RNA using HTS technology. The pooled sample was subjected to a preliminary quality check (QC) at Nucleome Biotech and the total RNA of the sample was quantified and qualified by using Agarose Gel Electrophoresis (1% Agarose Gel) and Qubit^®^ 3.0 Fluorometer (Thermo Fisher Scientific Inc.). One microgram total RNA was used for library preparation. Ribosomal RNA was removed from total RNA using Ribo-Zero Magnetic Kit (Epicentre, Madison, WI, USA). HTS library preparations were constructed according to the manufacturer's protocol (NEBNext II RNA Library Prep Kit for Illumina^®^). Ready-to-run final libraries were quantified using a Qubit 3.0 Fluorometer (Thermo Fisher Scientific Inc.) using a DNA HS assay kit (Thermo Fisher Scientific Inc.) following the manufacturer's protocol. To identify the insert size of the library, we queried it on TapeStation 4150 (Agilent) utilizing highly sensitive D1000 ScreenTape (Agilent) following manufacturer's protocol. Libraries were sequenced by Illumina high-throughput sequencer with paired-end sequencing strategy. The qualified libraries were fed into Illumina sequencers NovaSeq 6000, S4 Flow Cell (2 x 150bp Read Length) after pooling according to their effective concentration and expected data volume.

### Bioinformatic analysis

The Raw data from Illumina Sequencer was obtained in a FASTQ format which contains sequenced reads and corresponding sequencing quality information (Cock et al., 2010). For virome reconstruction and virus identification, the raw reads obtained from high-throughput sequencing were trimmed, and filtered to remove low-quality reads and adapter sequences using Fastp version 2.8 (Chen et al., 2018). Guanine–cytosine (GC) content, Phred quality scores (Q20 and Q30), and sequence duplication level were calculated. Reads that passed the quality filtering were used for downstream analysis. Clean reads were assembled de novo by using assembly software Trinity version 2.10.0 (Henschel et al., 2012), Spades (Prjibelski et al., 2020), and Rnabloom (Nip et al., 2020) with the default setting to create the complete genome assemblies. The taxonomic profiling of high-throughput sequencing of assembled contigs was performed by Kaiju version 1.7.3 (Menzel et al., 2016) using the NCBI reference sequence database and the Krona tool was used to visualize the results.

### Identification of viruses

The contigs obtained after de novo assembly were subjected to Blastn and Blastx of GenBank database (NCBI) (https://www.ncbi.nlm.nih.gov/) to confirm the viral/viriod sequences present in the assembled library. Contigs that mapped with the individual viral genome sequences from the NCBI were used to identify candidate viruses present in analyzed common bean samples. The open reading frame (ORF) of the identified viruses was found using the ORF finder NCBI (https://www.ncbi.nlm.nih.gov/orffinder/).

### Pairwise and phylogenetic analysis

The assembled viral genomes identified in this study from HTS were subjected to Blastn search of the NCBI database to retrieve the corresponding homologous genomes. Only the complete genomes that mapped with the assembled viral genomes in the present study were used to determine the pairwise identity and phylogenetic relationship. The genomes were trimmed to equal sizes and aligned using the ClustalW multiple alignment program in BioEdit version 7.2.5 (Hall, 1999). Pairwise nucleotide identities were calculated using the sequence demarcation tool (SDTv1.2) (Muhire et al., 2014) and identity scores were generated using color coded matrix. Maximum likelihood method was used to establish the phylogenetic relationship among the aligned sequences at 1000 replications for each bootstrap value in the MEGA X software (Kumar et al., 2018).

### Recombination analysis

Sequences used for pairwise and phylogenetic analysis were used for the detection of recombination events like the potential recombinant sequences, number of recombination events, major and minor parent, and recombination breakpoints, by using the recombination detection program (RDP) v.4.85 (Martin et al., 2015) with default settings. RDP uses different recombination detecting programs like RDP, GENECONV, Chimaera, MaxChi, BOOTSCAN, SISCAN, 3Seq, and LARD to generate evidence of recombination. Recombination events supported by three or more algorithms present in RDP with significant P-values were considered positive events.

### Data availability

Four near-complete viral genomes were deposited in GenBank, including two BCMVs (accession no. MW675689 and MW675688), one BCMNV (OK094708), and one ClYVV (MW675690).

### Confirmation of identified viruses by RT-PCR

The presence of viruses identified by HTS was confirmed initially by RT-PCR by extracting the total RNA from samples used for HTS library preparation. The complementary DNA (cDNA) synthesis was done using RevertAid First Strand cDNA Synthesis Kit (Thermo Fisher Scientific Inc.) following the manufacturer's instructions with slight modification. Specific primers for each virus were designed based on highly conserved regions using Primer3Plus software (https://www.bioinformatics.nl/cgi-bin/primer3plus/primer3plus.cgi). The RT-PCR was performed in 25 μl reactions containing 12.5 μl of GoTaq Green Master Mix (2X) (Promega), 2 μl of template cDNA, 1 μl of each forward and reverse primer (10 μM), and 8.5 μl of nuclease-free water. The PCR program used for all the viruses was the same with an initial denaturation step of 95°C for 2 minutes, followed by 35 amplification cycles of 95°C for 30 seconds, 55°C for 30 seconds, 72°C for 1 minute, and final extension at 72°C for 10 minutes. The amplicons were resolved on 1 % agarose gel (containing TAE buffer) with 1Kb ladder (Thermo Fisher Scientific Inc.) as molecular marker stained with ethidium bromide (Thermo Fisher Scientific Inc.) and first visualized on UV-transilluminator and then further analyzed on gel documentation system to capture gel images. The amplicons of each virus were sequenced for reconfirmation using Sanger sequencing (Biokart India Pvt. Ltd.).

### RT-PCR-based incidence of identified viruses

All the collected samples (140) maintained at −80 °C were used to determine the distribution and incidences of identified viruses in the Kashmir valley by using RT-PCR. Total RNA extracted from these samples was subjected to RT-PCR as explained above. RT-PCR-based incidence was calculated as the percentage of plant samples infected with viruses.

### Multiplex PCR

The multiplex PCR was optimized for the simultaneous detection of three identified viruses in this study. Samples having co-infection of all the identified viruses were used as positive control and healthy plant samples as a negative control for multiplex PCR. The total volume of the multiplex PCR reaction mixture was 25 μl containing four different primer sets and 12.5 μl GoTaq Green Master Mix (2X) (Promega). The primers used in uniplex PCR were also used in multiplex PCR. For the optimization and standardization of multiplex PCR, designed primer with different volumes from the working solution of 10μM concentrations (0.4, 0.5, 0.6, 0.8 μl) at varied annealing temperatures (50, 52, 55, and 58°C) was used. Amplification by multiplex PCR was performed at the initial denaturation step of 95°C for 2 minutes followed by 35 amplification cycles of denaturation at 95°C for 30 s, varied annealing temperatures (50, 52, 55, and 58°C) for 30 s, extension at 72°C for 1 min, and final extension temperature of 72°C for 10 min. The amplicon was electrophoresed in a 1% agarose gel (containing TAE buffer) with 1Kb ladder (Thermo Fisher Scientific Inc.) as a molecular marker run on 80 V for 1 h stained with ethidium bromide and visualized on UV transilluminator.

The total RNA isolated from infected plant samples had a concentration of 400 ng/ μl. To evaluate the sensitivity of multiplex PCR, the cDNA containing all the viruses was diluted 10-folds (10^−1^ to 10^−8^) using nuclease-free water. Each dilution was used as a template for PCR to determine sensitivity.

Finally, the multiplex PCR assay standardized was validated on several common bean samples collected from the field.

## Results

### Virus identification and transcriptome assembly

The pooled RNA passed the quality control (QC) and showed an RNA integrity number (RIN) of 7.3. To guarantee the reliability of the data, QC was performed at each step of the procedure. A total of 16.9 GB of sequence data was obtained from Illumina sequencing of constructed cDNA libraries with 111783988 raw reads and 16879382188 bases. After trimming, we obtained 12051150320 bases with quality scores of Q30 and 49% GC content. *De novo* assembly of clean reads was performed using three different assemblers Trinity, Spades, and RNABloom generated 39837, 37429, and 50704 sequences, respectively, with N50 values for contigs ranging from 558 to 835 bp.

**Figure 2:**
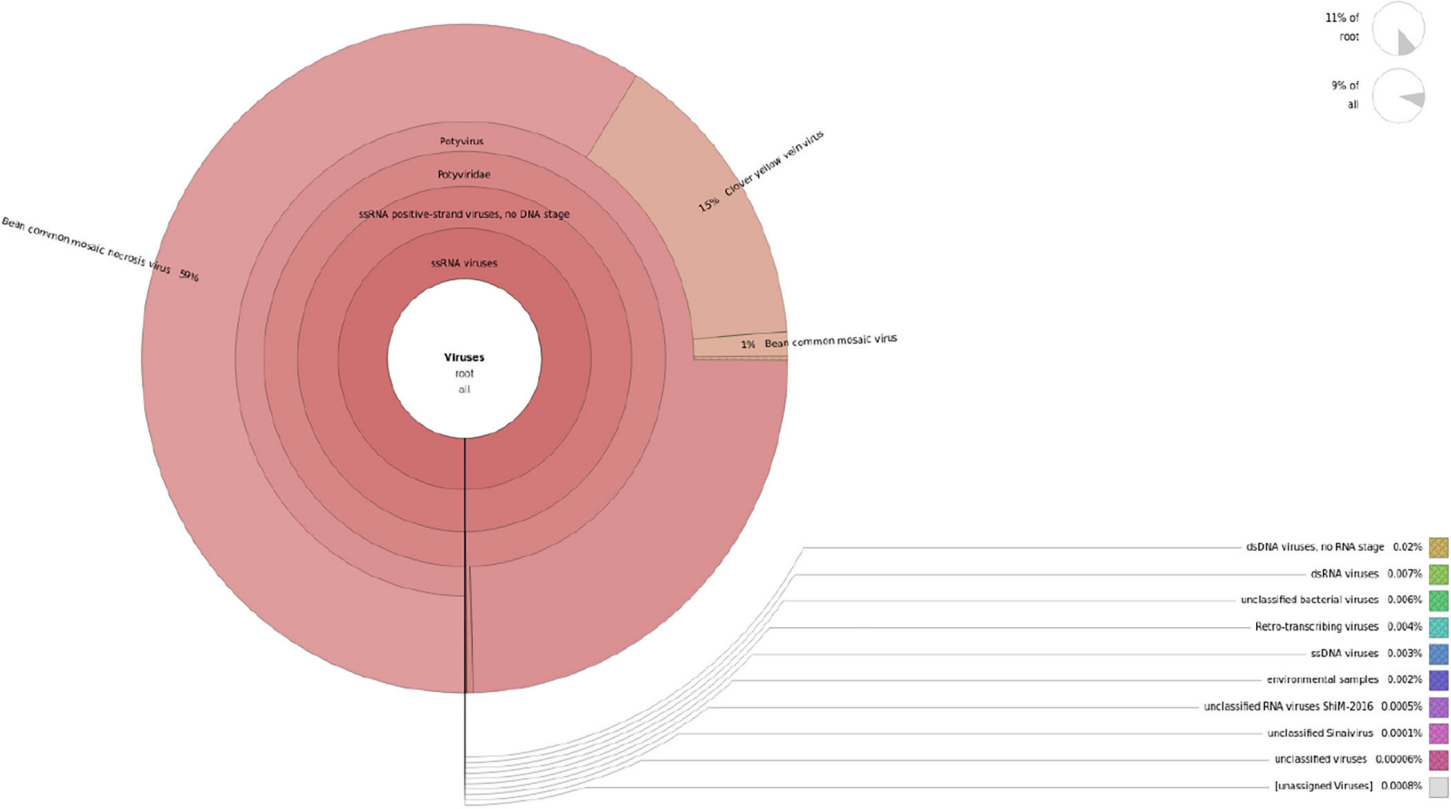
Krona representation of viral sequencing data obtained from Illumina.

The contigs assembled were blasted against the virome database to determine the virus-associated contigs. Contigs that showed similarities with plant viruses were identified as BCMV, BCMNV, and ClYVV all belonging genus potyvirus, family potyviridae. A total of 50,315 to 58,778 virus-associated sequences were identified from these three assemblers, in which 16,781, 17,007, and 19,571 assembled sequences of BCMV, 16,759, 17,000, and 19,614 assembled sequences of BCMNV, and 16,775, 16,999, and 19593 assembled sequences of ClYVV were generated by Trinity, Spades, and RNABloom, respectively (Table 1). Taxonomic classification results showed that the majority of viral sequences were derived from BCMNV, representing 59% of total viral reads. BCMV and ClYVV were represented by 1% and 15% of total viral reads, respectively (Figure 2). The de novo assembly and Blastn results generated 18 partial/near-complete genomes of these viruses, including 10 genomes of BCMNV with the length between 308 and 9,967 nt, six genomes of BCMV with a size of 324 to 9964nt, and three genomes of ClYVV with lengths in the range from 227 to 9,759 nt (Supplementary Table 1).

**Table 1:** Descriptive statistics of assembled viral reads.

	**Trinity**			**Spades**			**Rnabloom**		
Virus strain	CYVV	BCMV	BCMNV	BCMV	CYVV	BCMNV	BCMV	CYVV	BCMNV
num_seqs	16775	16781	16759	17007	16999	17000	19571	19593	19614
sum_len	8468024	8552674	8456149	6869645	6877704	6878116	7919227	7857367	7910617
min_len	181	181	181	184	184	184	200	200	200
avg_len	504.8	509.7	504.6	403.9	404.6	404.6	404.6	401	403.3
max_len	20380	18822	20380	18012	18012	20379	18012	15738	16959
Q1	246	247	247	226	226	226	231	232	232
Q2	325	325	325	283	283	283	282	284	284
Q3	519	519	517	422	422	422	401	402	399
N50	584	594	582	429	430	430	405	402	402

### RT-PCR confirmation of identified viruses

**Figure 3:**
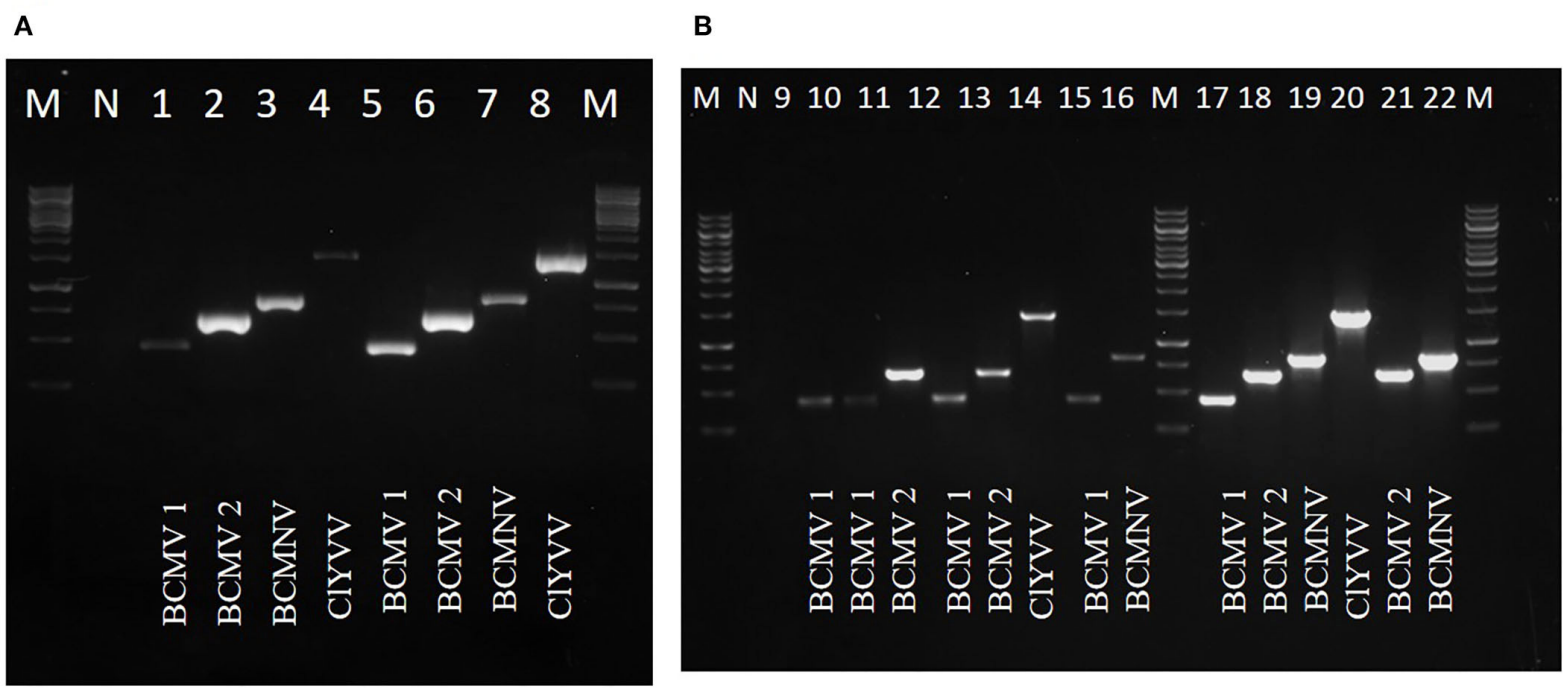
RT-PCR confirmation of identified viruses by using newly designed virus-specific primers and **(A,B)** represents amplicons of identified viruses from lane 1 to 22, BCMV 1 (442bp), BCMV 2 (661bp), BCMNV (834bp), and ClYVV (1442bp). M is 1Kb molecular marker. N is the negative control of the healthy plant.

Viruses identified by HTS were confirmed by RT-PCR using specific primers for each virus against samples used for RNA-Seq. Four virus-specific primers designed based on highly conserved regions were used (Table 2). The presence of all three viruses was confirmed with an expected amplicon size of 442bp (BCMV 1), 661bp (BCMV 2), 834bp (BCMNV), and 1443bp (ClYVV). RT-PCR amplified virus-derived fragments were assessed on agarose gel (Figures 3A,B) and further confirmed by Sanger sequencing. Sequencing results showed 100% similarities with the contig sequences derived from the HTS data. Both the RT-PCR and HTS results indicated that all the viruses identified by HTS were actually present in common bean samples sent for HTS sequencing.

**Table 2:** Primer designed and used in the present study.

**S. No**.	**Primer Name**		**Primer Sequences 5^′^-3^′^direction**	**Product Size (bp)**
1	BCMV1	F1	TAGCTCACTTGGGGAATTGG	442
		R1	TGGATCAACAAAACGGATCA	
2	BCMV2	F1	GATTCTGGAGTGGGACAGGA	661
		R1	ATACTCGCCCTTCACAGCAT	
3	BCMNV	F1	ATGAACAGTGTGGCGAAGTG	834
		R1	GCTTTGTTGGGCTCTTCAAC	
4	ClYVV	F1	CAGTGCACCCAAGTCATGAG	1443
		R1	ACCTCACTTAGCTACTCTGTCAG	

Seventy-nine out of 140 samples were found positive for these viral pathogens, with BCMV, BCMNV, and ClYVV being present in all 79, 38, and 23 samples, respectively (Table 3). None of the asymptomatic samples showed the presence of these viruses. The majority of common bean samples showed positive reactions for more than one virus. The mixed infection rate was observed highest between BCMV and BCMNV. Among 79 samples, mixed infection was present in 44 samples. The BCMNV and ClYVV were always associated with BCMV and not a single sample was found in which only BCMNV or ClYVV was present. These were always present with BCMV in mixed infections as BCMV+ BCMNV + ClYVV or BCMV + BCMNV or BCMV + ClYVV.

**Table 3:** Distribution of viruses in Kashmir using RT-PCR.

**S No**.	**Region**	**Districts**	**Site of** **Collection**	**No. of** **Samples** **Collected**	**No. of Samples Positive** **for** **Viruses**			**No. of Samples** **Negative for** **Viruses**	**No. of** **Multiple** **Infections**
					**BCMV**	**BCMNV**	**CYVV**		
1	South Kashmir	Anantnag	Doru	10	5	2	1	5	2
			Zalangam	10	3	0	0	7	0
2		Pulwama	Kakapora	10	5	3	1	5	4
			Tikan	10	4	2	0	6	2
3	Central Kashmir	Srinagar	Noor bagh	10	7	3	2	3	4
			SKUAST-K	10	9	7	5	1	8
4		Budgam	Budgam	10	8	4	3	2	4
			Panzan	10	6	3	2	4	4
5		Ganderbal	Lar	10	6	2	2	4	3
			Prang	10	7	3	3	3	3
6	North Kashmir	Baramulla	Wadura	10	5	3	2	5	3
			Logripora	10	3	1	0	7	1
7		Kupwara	Cherkyoot	10	6	3	1	4	4
			Lalpora	10	5	2	1	5	2
		Total		140	79	38	23	61	44

RT-PCR of common bean samples from the Kashmir valley revealed the incidence of 56.42% for BCMV, 27.14% for BCMNV, and 16.42% for ClYVV. The highest incidence of all three viruses was observed from the field of SKUAST-K in the district Srinagar of central Kashmir. Of the 140 samples collected from Kashmir, 43.57% samples had no viral infection. Results have also revealed that these viral pathogens were prevalent in all the different locations surveyed.

### Genome organization, pairwise nucleotide comparison, and phylogenetic analysis of detected viruses

**Figure 4:**
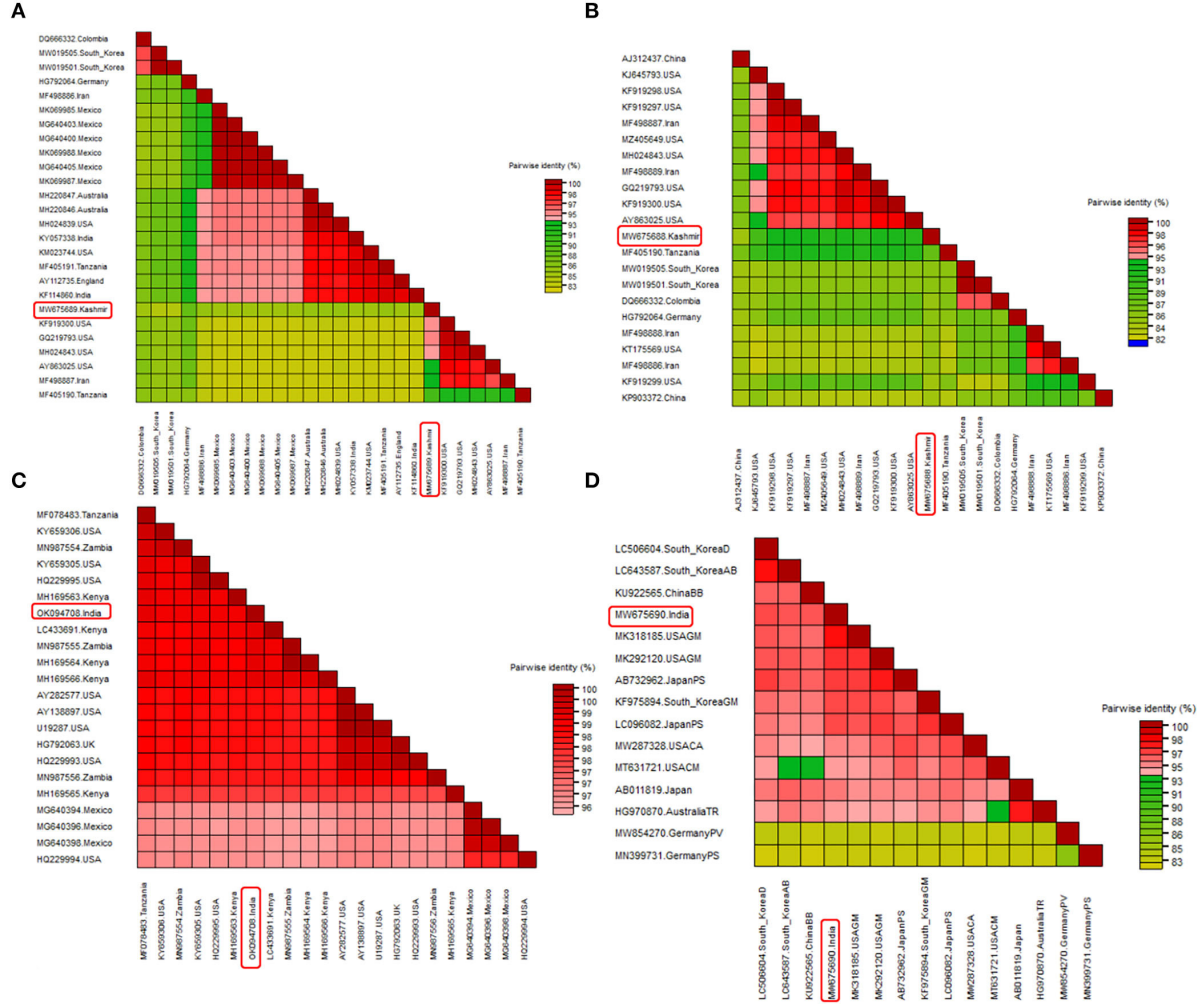
Pairwise nucleotide comparison of identified viruses along with closely related genomes obtained from GenBank NCBI database. Color coded matrix was generated using SDT. Each color in the matrix represents percent identity score. **(A)** Pairwise comparison of BCMV 1, **(B)** pairwise comparison of BCMV 2, **(C)** pairwise comparison of BCMNV, and **(D)** pairwise comparison of ClYVV.

**Figure 5:**
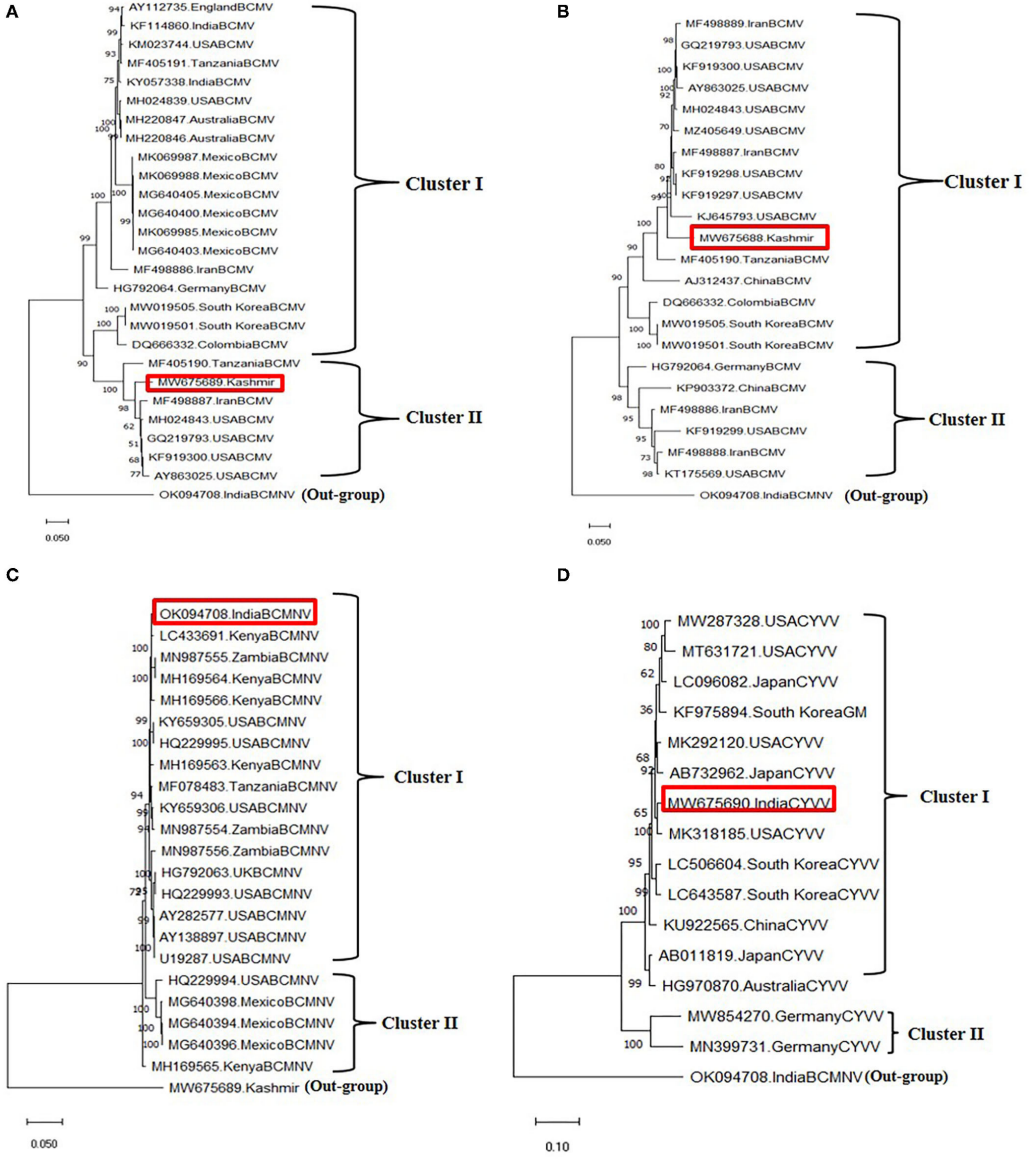
Phylogenetic analysis of identified viruses using whole genomes through maximum likelihood method using the MEGA X software. The scale indicates the number of substitutions per site. Highlighted in red in phylogeny represents the isolates of the present study. **(A)** Phylogenetic tree of BCMV 1, **(B)** phylogenetic tree of BCMV 2, **(C)** phylogenetic tree of BCMNV, and **(D)** phylogenetic tree ClYVV.

Two field BCMV isolates obtained from HTS were named BCMV 1 (MW675689) and BCMV 2 (MW675688), with genome sizes of 9964 and 9944 nt, respectively. The pairwise nucleotide identity between BCMV1 (Kash1) and BCMV2 (RU1) was 89%. The ORF of BCMV 1 comprises 9609 nt which encodes a predicted polyprotein of 3202 amino acids (aa). The two untranslated regions (UTR) in the 5' and 3' end of genomic RNA consisted of 127 nt and 238 nt, respectively (Table 4). The pairwise nucleotide identity of BCMV 1 with other similar BCMV isolates ranged from 83% to 94% (Figure 4A). The highest nucleotide identity of 94% was shared by sequences from the USA (KF919300, GQ219793, MH024843). The phylogenetic analysis showed a similar trend as indicated by pairwise analysis. The BCMV1 isolate clustered together with the six other BCMV isolates from the USA, Iran, and Tanzania in cluster III of the phylogenetic tree (Figure 5A).

**Table 4:** The genome size, organization, and accession numbers of viruses identified from common bean leaf samples from next-generation sequencing.

**Virus name**	**Genome size (Kb)**	**5' UTR (nt)**	**Start codon**	**Stop codon**	**3'UTR**	**ORF size (Kb)**	**No. of amino acids**	**Accession no**.
BCMV 1	10	127	128	9736	228	9.6	3202	MW675689
BCMV 2	10	125	126	9716	228	9.6	3196	MW675688
BCMNV	9.6	157	158	9373	228	9.2	3071	OK094708
ClYVV	9.8	286	287	9505	252	9.2	3072	MW675690

In case of BCMV 2, the single polyprotein encodes for 3196 amino acids from 9591 nucleotide long ORF with 127 nt 5' UTR and 238 nt 3' UTR (Table 4). The pairwise nucleotide comparison found that BCMV 2 isolate showed 83% to 92% identity score range with known isolates in GenBank. The sequences from the USA (KF919298, KF919297, MH024843, GQ219793, KF919300) and Iran (MF498887) presented 92% identity scores with BCMV 2 isolate (Figure 4B). Similar results were reflected in phylogenetic analysis where BCMV 2 isolate clustered with isolates from the USA and Iran in cluster I (Figure 5B). The polyprotein comparison of BCMV 1 and BCMV 2 with the first 50 protein sequences in the NCBI GenBank revealed the percent identity of 87–98% and 89–94 %, respectively. Conserved domains of both the isolates of BCMV were also determined (Supplementary Figures 1A,B).

The near-complete genome of BCMNV obtained from HTS consisted of 9601 nt. The genomic RNA of BCMNV contained a single long ORF that starts at 158 nt and terminates at 9373 nt, encoding a single polyprotein of 3071 aa and 157 nt 5' UTR and 228 nt 3' UTR (Table 4). The polyprotein of BCMNV showed a similarity of more than 97% when compared with all other polyproteins of BCMNV available on NCBI. The conserved domains in BCMNV encoded protein were determined by using the conserved domain database (CDD) NCBI. The graphical results of the CD search obtained for BCMNV representing the whole viral genome are shown in Supplementary Figure 2A. This is the first near-complete genome sequence of BCMNV from India. The BCMNV isolate of the present study shared 96–99% pairwise identity with the other BCMNV isolates from NCBI GenBank. The isolates from Kenya (LC433691, MH169564, MH169566) and Zambia (MN987555) shared the highest pairwise nucleotide similarity of 99% (Figure 4C). Similar results were evident in phylogenetic analysis, where the identified BCMNV isolate formed a tight cluster with the above sequences in cluster I (Figure 5C).

The identified ClYVV isolate contained a genomic RNA of 9757nt including 5' (286 nt) and 3' (252 nt) UTR. The single large ORF of 9219 nt encodes a polyprotein of 3072 aa (Table 4). The polyprotein comparison of ClYVV shared more than 92% similarity with all other ClYVV protein sequences present on NCBI. Also, the functional CD of ClYVV was determined (Supplementary Figure 2B). This is the first report of ClYVV from India in common beans. The pairwise identity using SDT revealed that the reference sequence of USA (MK318185) showed more than 97% pairwise nucleotide identity (Figure 4D). In addition, phylogenetic analysis indicates that ClYVV recovered in this study was grouped in cluster I along with isolate from USA (MK318185) (Figure 5D).

To establish the inter-species phylogenetic relationship, a combined maximum likelihood phylogenetic tree was constructed using the available nucleotide and polyprotein sequences of all three viruses (Supplementary Figure 3). The 74 whole-genome sequences formed three highly supported clusters (I, II, III) representing 37 BCMV isolates (I), 22 BCMNV isolates (II), and 15 ClYVV isolates (III). BCMV isolates exhibited high diversity and variability with two sub-clusters (a and b) being classified. BCMV 1 and 2 were clustered closely in sub-cluster b along with genomes from Iran, the USA, China, and Tanzania. Also, the polyprotein sequences formed three clusters separating three viruses. But in contrast to nucleotide sequences, the two polyprotein sequences of BCMV isolates formed two separate sub-clusters (Supplementary Figure 4).

### Recombination analysis

**Figure 6:**
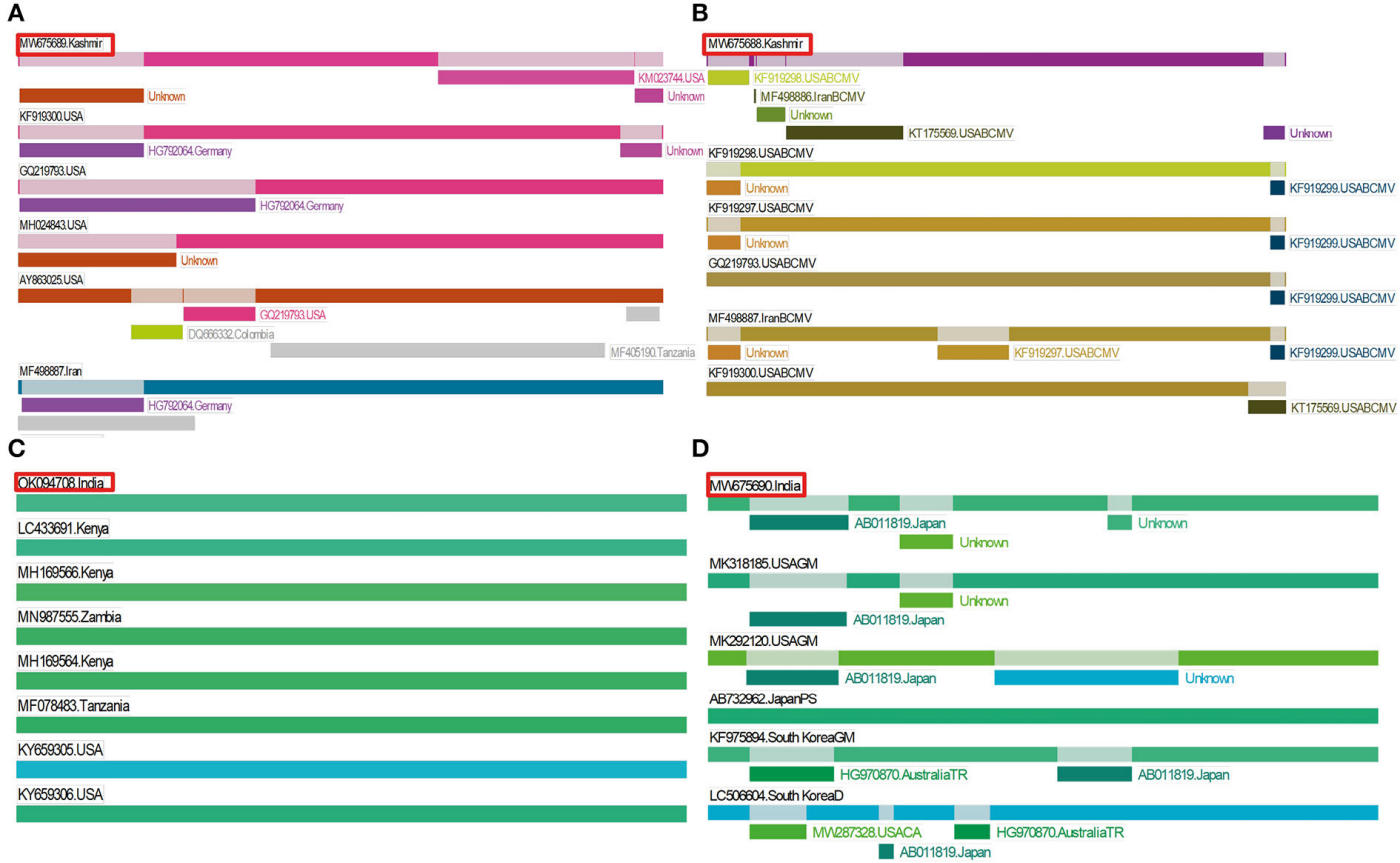
Recombination events detected in identified viruses by using RDP. Position of our isolate is highlighted in red color. **(A)** Three recombination events detected in BCMV 1, **(B)** four recombination events detected in BCMV 2, **(C)** recombination was not found in BCMNV, and **(D)** two recombination events detected in ClYVV.

In potyviruses, recombination occurs frequently and is one of the major drivers that may affect the evolution of potyviruses (Zhou et al., 2014). The recombination events were detected in two isolates of BCMV and ClYVV identified in the current study. These recombination events were detected by three or more algorithms with associated P-values of ≤1.0x10^−5^. However, no traces of recombination were observed in the BCMNV isolate of the present study (Table 5, Figure 6).

**Table 5:** The recombination events detected along with major and minor parents, p-value of viruses detected in common bean.

**Virus** **Name**	**Event**	**Recombinant**	**Major** **parent**	**Minor** **parent**	**Break** **point** **start**	**Break** **point** **end**	**Methods**						
							**RDP**	**BOOTSCAN**	**GENECONV**	**MAXCHI**	**CHIMAERA**	**SISCAN**	**3Seq**
							**P-value**						
BCMV 1	1	MW675689	HG792064 (Germany)	Unknown	20	1,947	2.278×10^−210^	1.726 × 10^−82^	1.154 × 10^−265^	2.644 × 10^−52^	3.477 × 10^−18^	2.513 × 10^−35^	1.927 × 10^−10^
	2	MW675689	KF919300 (USA)	KM023744 (USA)	6,446	9,468	6.226 × 10^−134^	1.025 × 10^−133^	3.612 × 10^−130^	5.988 × 10^−44^	4.076 × 10^−45^	3.635 × 10^−52^	4.425 × 10^−53^
	3	MW675689	MW019505 (South Korea)	Unknown	9,537	9,911	1.238 × 10^−24^	–	6.008 × 10^−21^	–	1.153 × 10^−06^	2.937 × 10^−17^	^2.247 ×^ 10^−13^
BCMV 2	1	MW675688	Unknown	KF919298 (USA)	32	748	1.082 × 10^−10^	3.769 × 10^−05^	3.010 × 10^−07^	4.346 × 10^−13^	3.150 × 10^−09^	3.628 × 10^−15^	1.476 × 10^−13^
	2	MW675688	MH024843 (USA)	Unknown	868	1,363	4.386 × 10^−70^	7.988 × 10^−64^	2.657 × 10^−67^	2.860 × 10^−21^	1.557 × 10^−21^	2.024 × 10^−19^	4.429 × 10^−13^
	3	MW675688	KJ645793 (USA)	KT175569 (USA)	1,364	3,420	1.948 × 10^−118^	5.766 × 10^−106^	1.234 × 10^−99^	7.000 × 10^−36^	1.632 × 10^−20^	8.279 × 10^−33^	1.476 × 10^−13^
	4	MW675688	MF498888 (Iran)	Unknown	9,627	10,011	1.311 × 10^−24^	4.481 × 10^−24^	7.536 × 10^−27^	1.337 × 10^−09^	8.326 × 10^−07^	4.359 × 10^−15^	1.476 × 10^−13^
**BCMNV**	**No Recombination**												
ClYVV	1	MW675690	AB732962 (Japan)	AB011819 (Japan)	567	1,938	6.739 × 10^−06^	2.050x10^−06^-	–	5.113 × 10^−09^	1.869 × 10^−08^	3.070 × 10^−10^	-
	2	MW675690	LC506604 (South Korea)	Unknown	2628	3,389	–	5.789 × 10^−05^	–	1.009 × 10^−05^	4.730 × 10^−05^	–	^−^

### Multiplex RT-PCR of identified viruses

**Figure 7:**
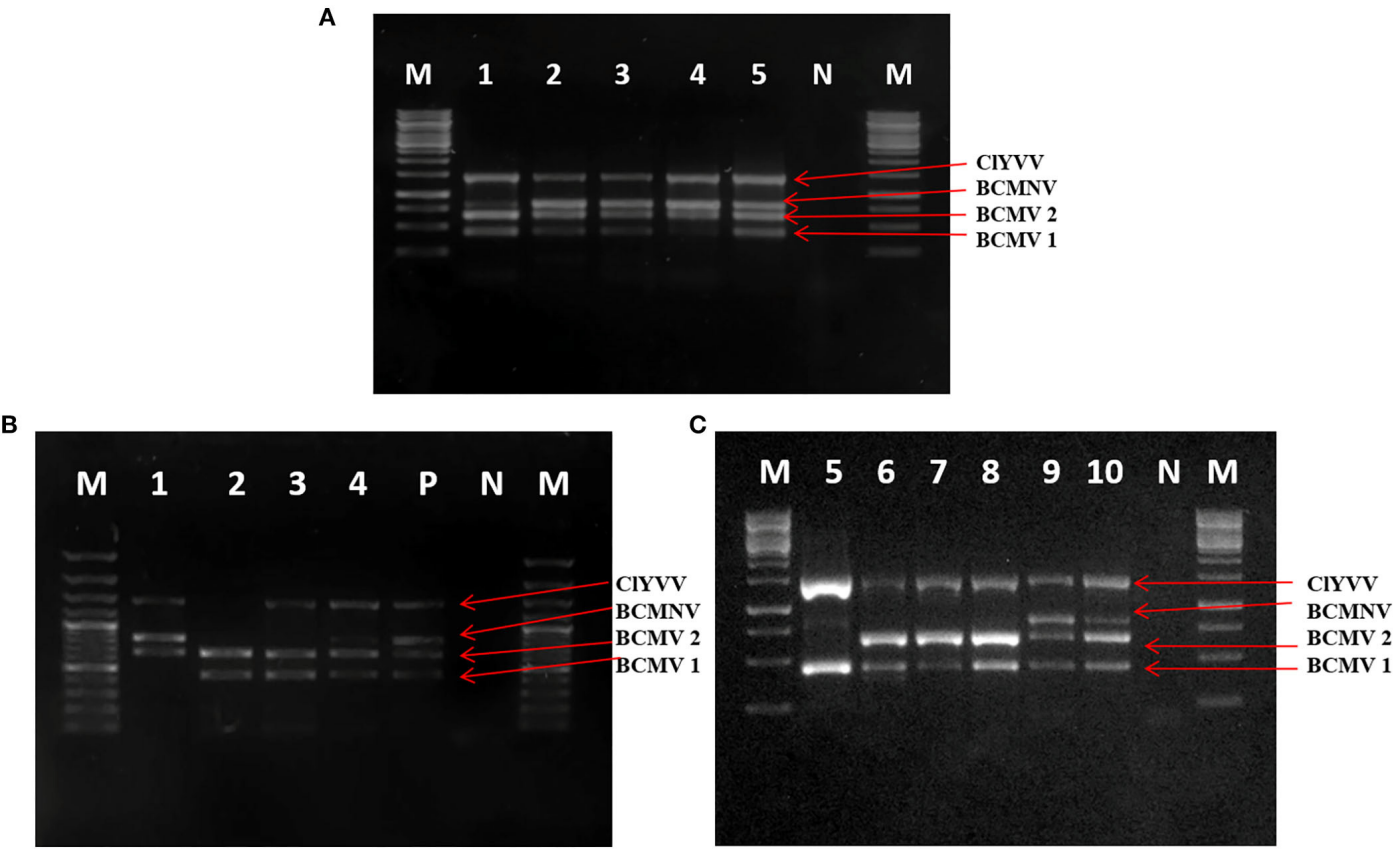
Optimization of multiplex PCR using specific primers of BCMV 1, BCMV 2, BCMNV, and ClYVV in RT-PCR assay. **(A)** Lane 1–5: PCR product using RNA with BCMV 1, BCMV 2, BCMNV, and ClYVV infection from positive control as a template for assay. **(B,C)** Simultaneous detection of bean viruses in multiplex RT-PCR by using RNA extracted from 10 field samples collected from SKUAST-K. Lanes 1–10 are amplified products of representative field samples. M is 1Kb molecular marker, P is positive control obtained from infected plants with all three viruses, and N is a negative control from a healthy plant.

It is important to develop the molecular diagnostics method for the detection of viruses infecting common beans. For that, specific primers were designed against the identified viruses and the multiplex PCR was standardized. Samples with multiple infections confirmed by uniplex PCR (positive control) were used for the optimization of multiplex PCR assay. The various experiments showed that when different annealing temperatures ranging from 50°C to 58°C were used, optimum results with no unspecific bands were obtained at 55°C with 0.4 μl from a working solution of 10μM concentration of each primer pair. The designed primers successfully generated their respective amplicons only from infected samples. In case of cDNA from a healthy plant, no amplicon was observed. The results of multiplex PCR were visualized by using gel electrophoresis on the gel documentation system (Figure 7A).

The detection limit of multiplex PCR using a 10-fold serial dilution of cDNA showed positive results up to a dilution of 10^−5^ for both BCMV and ClYVV and 10^−4^ for BCMNV. All three viruses were detected simultaneously by multiplex PCR up to a dilution of 10^−4^. To test the consistency and reproducibility of results with identical conditions, multiplex PCR was validated on several common bean samples infected with these viruses. All the viruses were identified from tested samples and the expected amplified fragments were visualized on 1% agarose gel (Figures 7B,C).

## Discussion

Viral metatranscriptomics along with bioinformatic tools has revolutionized plant virus discovery, detection, genome sequencing, transcriptomics, ecology, and epidemiology (Barba et al., 2014). The application of HTS to plant viral diagnostics has resulted in a significant increase in the number and frequency of new viruses detected. This new technology not only detects viruses of known sequences but also identifies untargeted viral pathogens regardless of their genomic nature or structure (Ibaba and Gubba, 2020). Virome analysis investigates all the viral genomes present in plant tissues, as well as their complexity, replication, mutation, and changes in response to diverse environments (Jo et al., 2020). Recently, several HTS-based studies have intensively explored common beans infected by viruses in different countries (Nordenstedt et al., 2017; Mutuku et al., 2018; Mwaipopo et al., 2018; Alves-Freitas et al., 2019).

Our goal in this study was to survey common bean fields in Kashmir for virus infection and to investigate molecular variability and genetic diversity of identified viruses by using HTS and downstream molecular techniques. Symptomatic as well as non-symptomatic samples were collected in our study to ensure that potential cryptic viruses would not escape from detection. Sample pooling is an efficient way to balance virus identification and cost reduction. Ribosomal RNA-depleted total RNA was used for virome analysis. This method has been used successfully to detect both RNA and DNA viruses (Gaafar et al., 2020). Among the three viruses we identified, neither BCMNV nor ClYVV has been documented to occur in common bean fields of India before. BCMV occurrence in Kashmir was previously reported by Hamid et al. (2013). Our work represents the first viral metatranscriptomics study of infected common beans in India.

Mixed infection of plants by different viruses occurs commonly in nature as the consequence of successive vector inoculations (Rubio et al., 2020). We observe the association of BCMV to BCMNV or ClYVV infection in our study, indicating that mixed infections are not in the manner of random combinations. Some symptomatic samples in our study turned out to be RT-PCR negative. Their virus-infection-like symptoms could be due to nutrient deficiencies, deformities, and/or other injuries (Chiquito-Almanza et al., 2017). Fungal or bacterial infection of vascular tissues and roots may also resemble virus-caused symptoms (Nordenstedt et al., 2017). Most of the virus-infected samples were from the district of Srinagar, which could be due to the vector population difference affected by the altitudes (Myers et al., 2000). Consistent with previous surveys in India (Yaraguntaiah and Nariani, 1963) and particularly in Kashmir (Hamid et al., 2013), BCMV is the predominant common bean-infecting virus in Kashmir as revealed by our latest surveillance. BCMNV and ClYVV could be introduced just recently.

The genome sizes (~10 Kb) of the three viruses identified were similar to those of other potyviruses (Takahashi et al., 1997; Worrall et al., 2015). The pairwise nucleotide identities of potyvirus genomes from different species are generally lower than 76% (Adams et al., 2005). A sequence similarity greater than this value represents different isolates of the same species. Unlike BCMV or ClYVV, the BCMNV isolate we identified has less than 10% divergence from other known isolates. This is consistent with previous studies detecting low diversity among BCMNV isolates (Collins et al., 2019). The combined phylogenetic analysis detects higher diversity in BCMV isolates, which is also consistent with the previous study (Worrall et al., 2015). The similarity and close clustering of our identified viruses with the isolates from the USA, Kenya, Tanzania, and Iran indicate that these viruses might have been introduced from the countries mentioned above (Collins et al., 2019; Sidharthan et al., 2020).

In potyviruses, recombination frequently occurs to serve as an important source of genetic variation (Seo et al., 2009; Zhou et al., 2014). Recombination also drives the genesis of new viral strains (Zhou et al., 2014; Worrall et al., 2015). Recombination rates in individual potyviruses could differ significantly (Revers et al., 1996). Feng et al. (2014) recently described a new recombinant strain RU-1M of BCMV, which causes temperature-independent necrosis in common bean plants harboring dominant I gene and recessive bc1 resistant gene. This study indicates that recombination may increase the threat of overcoming host resistance. The recombination events found in our BCMV and ClYVV isolates may facilitate their spread in Kashmir. Despite previously reported recombination in BCMNV (Larsen et al., 2005; Wainaina et al., 2019), no trace of recombination is found in the BCMNV isolate we reported in our study. The absence could be attributed to the founder effect caused by the initial introduction of low-diverse BCMNV variants to Kashmir (Seo et al., 2009).

Previous studies have shown the effectiveness of multiplex PCR in the simultaneous detection of three (Chiquito-Almanza et al., 2017), six (Cating et al., 2015), seven (Kwon et al., 2014; Zhao et al., 2015), eight (Kwak et al., 2014), and up to nine (Gambino, 2015) pathogens. The purpose of our optimization of multiplex PCR was to develop a robust, flexible, and accurate diagnostic tool for the detection of the identified viruses, as they produce similar symptoms and can be found within their hosts in multiple co-infections. Parameters posing challenges in multiplex PCR range from annealing temperature to master mix ingredients. Sensitivity of multiplex PCR was determined by the dilution of cDNA and all the viruses were amplified up to 10^−4^ dilution, indicating that these viruses can be detected simultaneously at lower concentrations of template cDNA. The optimized multiplex PCR assay developed in the present study can be used in virus diagnostic assays, seed certification, and crop improvement programs to obtain virus-free seed stocks.

The host resistance has been found to be the most efficient and economical option for managing the viral disease (Wagara and Kimani, 2007). In the present study, we found that common beans are infected by multiple viruses simultaneously, so the dominant I gene present in some bean cultivars may not be enough in conferring resistance against these viruses, being susceptible to BCMNV. The recessive resistant alleles available are strain specific to these viruses and, therefore, to provide broad resistance to the different strains of BCMNV and BCMV it is difficult to breed common bean cultivars using these genes individually. Thus, molecular markers can be used for pyramiding recessive genes (bc-u, bc-1, bc-1^2^, bc-2, bc-2^2^, and bc-3) along with the dominant I gene in order to develop the broadest resistance possible (Pasev et al., 2014).

### Contribution to the field

The present study has shown that HTS assay is a valuable and powerful tool for the detection and identification of virome in common beans. This diagnostic approach has superior accuracy and sensitivity, which enables early identification of viral diseases, critical for limiting their spread. In our study, we utilized HTS-based method and identified three potyviruses from common beans, with BCMNV and ClYVV being reported for the first time from India. BCMV was found most prevalent virus followed by BCMNV and ClYVV. Since all three viruses are aphid transmitted in a non-persistent manner and BCMV and BCMNV are also seed transmitted, these viruses have the potential to cause epidemic along with vector transmission and can emerge as major constraints in bean production. So, there is a need to establish efficient control measures in developing countries to limit the spread and transmission of these identified viruses. We also developed a multiplex PCR protocol that can be used for the detection and diagnosis of these viruses and can also aid in seed certification, quarantine, and crop improvement programs to produce virus-free seed material. Our HTS-based viral metatranscriptomics analysis adds genomic information on viruses infecting beans, thereby enabling rapid response to emerging viral diseases of this crop.

## Data availability statement

The datasets presented in this study can be found in online repositories. The names of the repository/repositories and accession number(s) can be found below: https://www.ncbi.nlm.nih.gov/, MW675689, MW675688, OK094708, and MW675690.

## Author contributions

Conceptualization: AH. Methodology, software, writing-original draft preparation, and visualization: AH and SR. Validation: AH, SR, and ZD. Formal analysis: AH, SR, and GA. Investigation: AH, SR, GA, TS, and ZD. Resources, supervision, and funding acquisition: AH and ZD. Data curation: AH and HP. Writing–review and editing: AH, SR, GA, TS, and ZD. Project administration: AH. All authors contributed to the article and approved the submitted version.

## Funding

This work has been funded by ICAR-IISS, Mau.

## Conflict of interest

The authors declare that the research was conducted in the absence of any commercial or financial relationships that could be construed as a potential conflict of interest.

## Publisher's note

All claims expressed in this article are solely those of the authors and do not necessarily represent those of their affiliated organizations, or those of the publisher, the editors and the reviewers. Any product that may be evaluated in this article, or claim that may be made by its manufacturer, is not guaranteed or endorsed by the publisher.
