# Supplementary material for: Viral metatranscriptomic approach to study the diversity of virus(es) associated with Common Bean (Phaseolus vulgaris L.) in the North-Western Himalayan region of India
Source: Front Microbiol. 2022 Sep 21;13:943382. doi: 10.3389/fmicb.2022.943382 (PMC9532741; doi:10.3389/fmicb.2022.943382)
Supplement: Supplementary file 1 [file Table_1.DOCX]

Supplementary table 2. Assembled viral contigs obtained from Trinity

|  | **Trinity BCMNV** | **Trinity bcmv** | **Trinity clyvv** |
| --- | --- | --- | --- |
| num_seqs | 10 | 6 | 3 |
| sum_len | 69972 | 21732 | 10425 |
| min_len | 308 | 324 | 227 |
| avg_len | 6997.2 | 3622 | 3475 |
| max_len | 9967 | 9964 | 9759 |
| Q1 | 310 | 326 | 227 |
| Q2 | 9810 | 586 | 439 |
| Q3 | 9946 | 9946 | 9759 |
| N50 | 9944 | 9946 | 9759 |
