# Supplementary figures and images for: Viral metatranscriptomic approach to study the diversity of virus(es) associated with Common Bean (Phaseolus vulgaris L.) in the North-Western Himalayan region of India
Source: Front Microbiol. 2022 Sep 21;13:943382. doi: 10.3389/fmicb.2022.943382 (PMC9532741; doi:10.3389/fmicb.2022.943382)

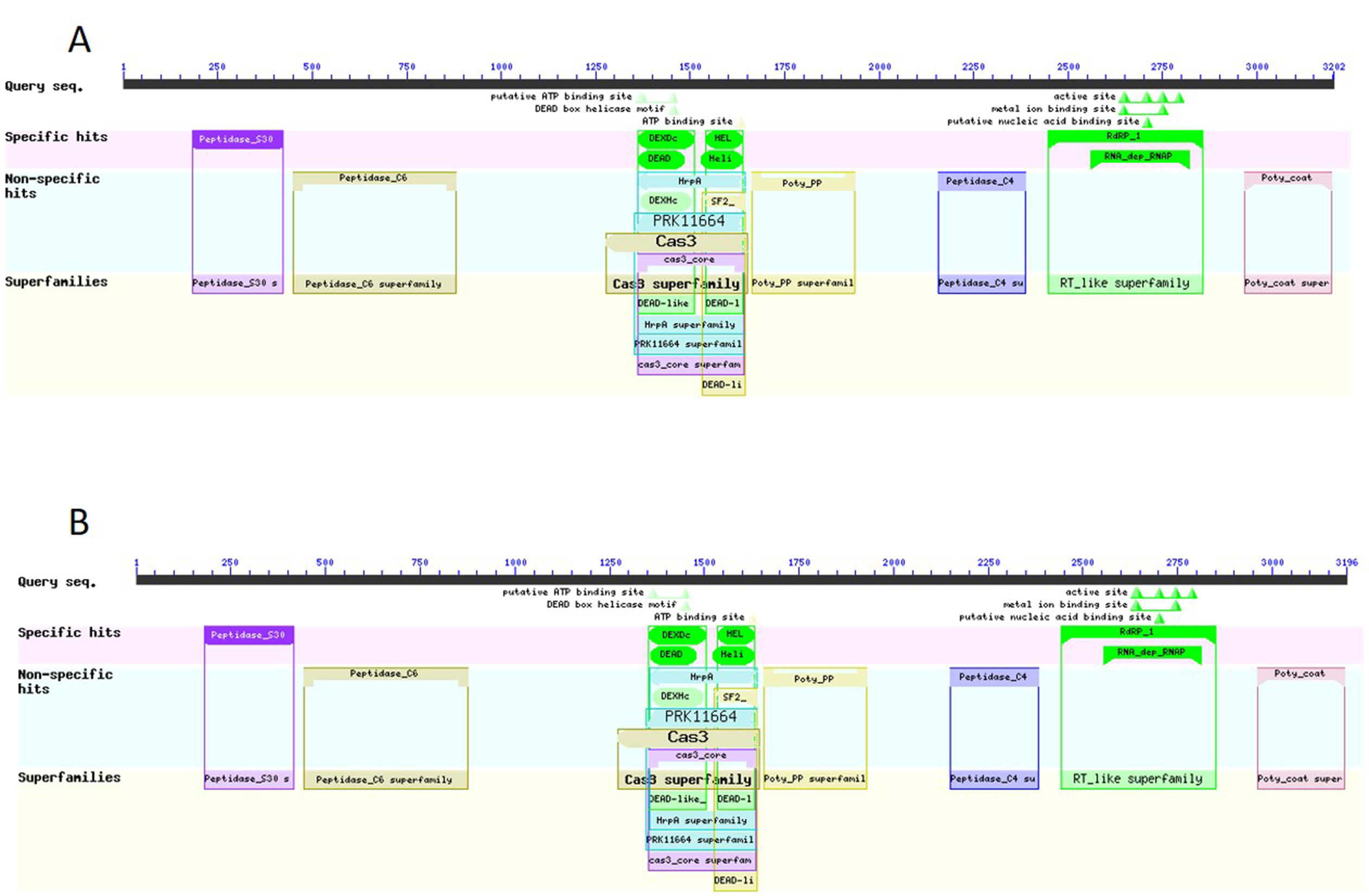

Supplement: Supplementary file 2 [file Image_1.JPEG]

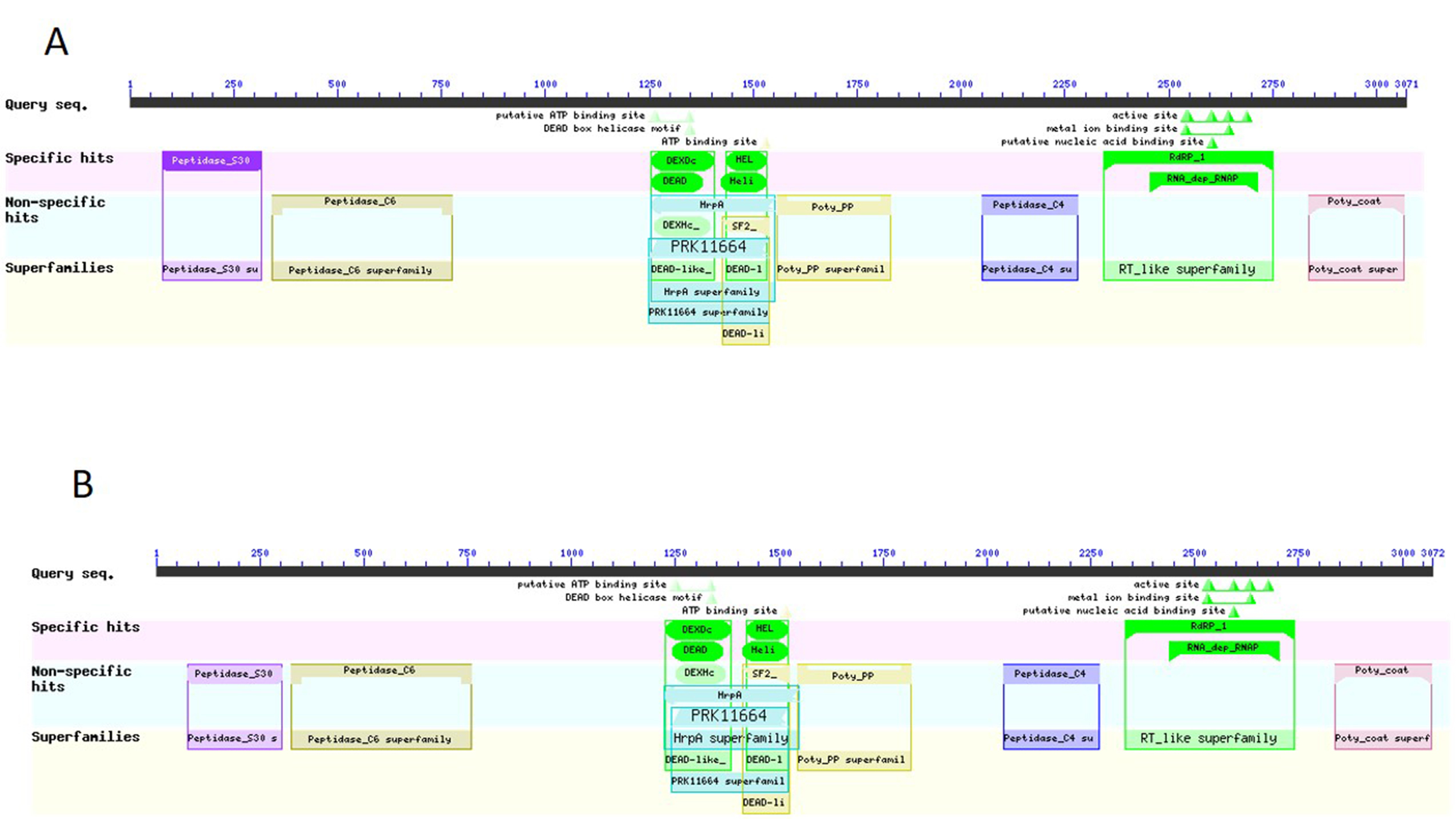

Supplement: Supplementary file 3 [file Image_2.JPEG]

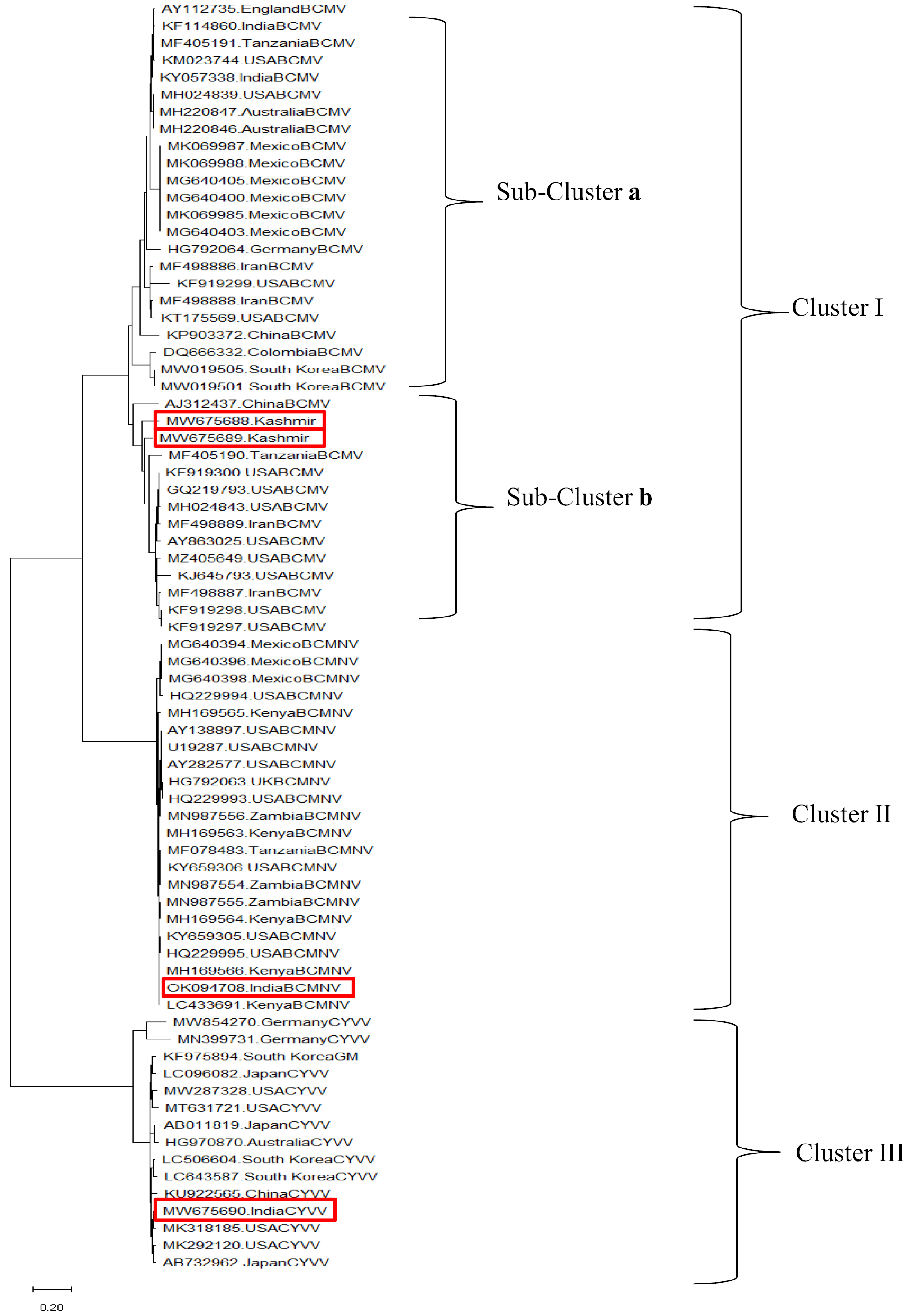

Supplement: Supplementary file 4 [file Image_3.TIF]

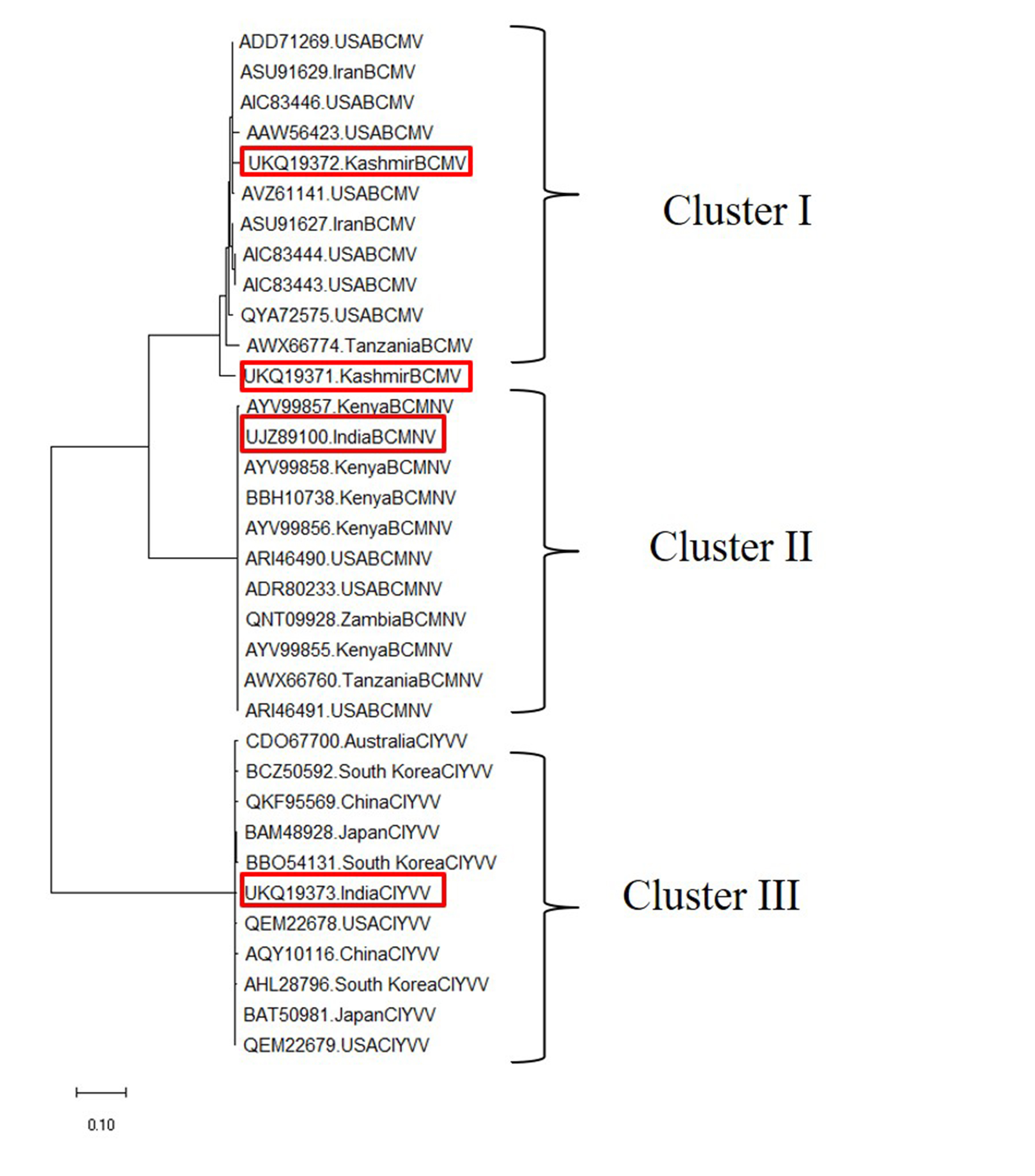

Supplement: Supplementary file 5 [file Image_4.JPEG]
